# Antifungal activity of the antimicrobial peptide RP557 against priority fungal pathogens

**DOI:** 10.1099/mic.0.001663

**Published:** 2026-03-02

**Authors:** Vanice Rodrigues Poester, Melissa Orzechowski Xavier, Jéssica Estefania Dávila Hidalgo, Mônica Campos dos Santos, Mariana Rodrigues Trápaga, Abdullah M.S. Al-Hatmi, Jesse Jaynes, David A. Stevens

**Affiliations:** 1 Mycology Laboratory, Post-Graduate Program in Health Science, Faculty of Medicine, Federal University of Rio Grande, Rio Grande, Rio Grande do Sul, Brazil; 2 Natural and Medical Sciences Research Center, University of Nizwa, Nizwa, Oman; 3 Colleges of Agriculture and Arts & Sciences, Tuskegee University, Tuskegee, Alabama, USA; 4 Div. of Infectious Diseases and Geographic Medicine, Stanford University Medical School, Stanford, California, USA

**Keywords:** *Aspergillus* spp., *Candida auris*, chromoblastomycosis and mycetoma species, *Cryptococcus neoformans*, dermatophytes, *Sporothrix *spp.

## Abstract

**Background.** Natural host defence molecules, part of innate immunity and the first line of defence, are evolutionarily conserved. Some pharmaceutical properties undesirable for clinical use led to the rational design of synthetic molecules with constructed peptide arrangements, giving a novel therapeutic avenue. A prior publication showed synthetic peptide RP557 inhibition and killing of fluconazole-sensitive and resistant *Candida* species isolates, biofilm inhibition, no resistance induction, direct membrane action, negligible mammalian cell toxicity and topical efficacy in a rodent vaginal candidiasis model. These findings highlight the relevance of investigating RP557 activity against other fungal pathogens.

**Objective.** We evaluated the antifungal spectrum of the RP557 against World Health Organization-listed priority fungal pathogens, including endemic and skin fungal pathogens, both alone and in combination with commercial antifungal drugs.

**Methods.** The antifungal spectrum was evaluated by broth dilution vs. clinical isolates, and we present 76 MICs (mcg ml^−1^) performed according to M27 or M38 CLSI documents, 35 checkerboard interactions with antifungals and 10 minimum fungicidal determinations.

**Results.** Overall impression is robust activity vs. chromoblastomycosis and mycetoma species, *Cryptococcus neoformans* and *Trichophyton* spp.; broad MIC ranges within most species, least activity vs. *Mucorales* and *Aspergillus* spp.; and some promising drug interactions vs. *Sporothrix* spp. and *Candida auris*.

**Conclusion.** Additional efficacy data *in vivo* is needed. Topical therapy could give local concentrations exceeding MICs, and burn or trauma prophylaxis or treatments are attractive potential targets owing to RP557 panmicrobial properties.

## Introduction

The global emergence of fungal pathogens, coupled with increasing resistance to available treatments, presents an important challenge to public health [1]. Additionally, the limited availability of effective drugs for opportunistic and endemic mycoses and the emergence of drug resistance further exacerbate this issue [2]. In response, the World Health Organization (WHO) has designated certain fungal pathogens as priorities for research, drug development and public health interventions, emphasizing the urgent need for new treatments and resistance studies. Among these, *Aspergillus* spp., *Cryptococcus neoformans*, *Candida auris*, *Candida albicans*, *Fusarium* spp., *Mucorales* and eumycetoma causative agents have been classified as critical or high-priority pathogens [3]. (Note: it has recently been advised [4] to reclassify *Candida auris* as *Candidozyma auris* going forward.)

Besides these agents of invasive fungal infections, skin mycoses caused by dermatophytes have a consequential impact on the quality of life for ~1.4 million patients worldwide each year [5]. Additionally, subcutaneous endemic mycoses caused by previously neglected pathogens such as *Sporothrix* spp. poses a severe but often underestimated public health challenge in several countries and continents [6,7]. Moreover, an explosive outbreak of sporotrichosis by *Sporothrix brasiliensis* has occurred in Brazil [8] and is spreading to adjacent and distant countries [9].

 In the search for new antifungal drugs, natural peptides represent a promising field of study. Natural compounds produced during the innate immune response of host organisms offer the advantage of low toxicity and have shown potential in reducing drug resistance in pathogens [10]. These molecules are highly evolutionarily conserved and are present in microbes, plants and all vertebrates. Moreover, in addition to direct antimicrobial effects, many members of this class of molecules have also been shown to stimulate host antimicrobial immunity [11,13]. A general term for them might be ‘host defence molecules’. However, some undesirable pharmaceutical properties (toxicity, solubility, unacceptable pharmacokinetics, stability and/or difficulty in manufacturing) have limited their clinical use, prompting the rational design of synthetic molecules with optimized peptide arrangements, including some peptides, and even some amino acids, not found in nature [14]. These synthetic approaches can improve half-lives *in vivo*, stability, solubility and resistance to host proteases. This approach has opened new therapeutic avenues, resulting in compounds with activity against bacteria, parasites, viruses and fungi [15].

 The synthetic antimicrobial peptide (AMP) RP557, a 17-aa compound, was selected for study after screening of several AMPs for antimicrobial activity. Previous studies showed its effectiveness in inhibiting and killing 46 fluconazole-sensitive and -resistant *Candida* isolates while also reducing preformed biofilm and inhibiting biofilm formation [16]. Additionally, this AMP does not induce resistance in *Candida* isolates, acts directly on the cell membrane (demonstrated by scanning electron microscopy), exhibits limited toxicity to mammalian cells and demonstrates topical efficacy in a rodent vaginal candidiasis model, comparable to miconazole [16]. Thus, we aimed to evaluate the antifungal spectrum of the AMP RP557 against WHO-listed priority fungal pathogens, as well as against endemic and skin fungal pathogens, both alone and in combination with commercial antifungal drugs.

## Methods

### Isolates and drugs

A total of 76 recent clinical isolates were used in this study, including *Candida auris* (*n*=6) (South Asian clade I) [17], *Cryptococcus neoformans* (*n*=5), *Aspergillus* spp. (*n*=17; *Aspergillus* section *Fumigati n*=13, *Aspergillus* section *Nigri n*=2, *Aspergillus* section *Flavi n*=2), *Fusarium* spp. (*n*=6), dermatophytes (*n*=5; *Trichophyton interdigitale n*=4, *Trichophyton rubrum n*=1), *Mucorales* (not further speciated; *n*=7), chromoblastomycosis and mycetoma pathogens (*n*=4; *Phialophora verrucosa, Cladophialophora carrionii*, *Fonsecaea pedrosoi* and *Madurella mycetomatis*, one isolate each) and *Sporothrix* spp. (*n*=26; *Sporothrix brasiliensis n*=20, *Sporothrix schenckii n*=5, *Sporothrix globosa n*=1). The isolates were obtained from fungal collections from the California Institute for Medical Research in San Jose, CA, USA, and the Laboratory of Mycology at Federal University of Rio Grande in Southern Brazil.

RP557 was synthesized by AmbioPharm (North Augusta, SC) using a synthetic solid-phase peptide synthesis scheme, with a purity of >96%, as confirmed by HPLC/mass spectrometry [16]. The molecular weight of RP557 is 2,140.75 daltons. The values shown in this study, as the concentrations for RP557, can be adjusted upward, in terms of true drug potency, as 15% of the concentrations shown in mcg ml^−1^ represent the weight of the acetate salt. Antifungal drugs were commercially obtained: itraconazole (ITZ) (Sporanox^®^, Janssen, Beerse, Belgium), fluconazole (FCZ) (Isofarma^®^, Ceará, Brazil), voriconazole (VCZ) (Accord Farmacêutica, São Paulo, Brazil), caspofungin (CAS) (Merck^®^, Rahway, New Jersey, USA), micafungin (MYC) (Sigma-Aldrich^®^, Missouri, USA) and amphotericin B (AmB) (Sigma-Aldrich). Drugs were tested in twofold dilutions, in concentrations as follows: RP557 from 0.5 to 64 µg ml^−1^; MYC, CAS, ITZ and VCZ from 0.03 to 8 µg ml^−1^, AmB from 0.06 to 16 µg ml^−1^; and FCZ from 0.25 to 64 µg ml^−1^. ITZ, VCZ and AmB were diluted initially in DMSO, while MYC, CAS, FCZ and RP557 were diluted in sterile water. All drugs were stored as stock solutions at 100×their final *in vitro* concentrations. All drugs and solutions were stored at 4 °C.

### Activity alone

 RP557 antifungal activity was evaluated following the Clinical and Laboratory Standards Institute (CLSI) protocols M27-Ed4 for yeasts and M38-A2 for moulds [18,19]. This is described in brief in this paragraph, as follows. The standardized inoculum was prepared according to each fungal requirement outlined in those protocols. All inocula were diluted in RPMI 1640 (a fully defined medium that would also allow microbial susceptibility testing in the future in the presence of mammalian cells), and the pour-plate technique was used to confirm the inoculum concentration as described. Incubation was carried out according to the CLSI protocol for each fungal species (for slow-growing mycelial fungi, readings were taken when growth in the absence of drug was deemed 4+), and the endpoint reading was as recommended for each drug per those protocols. Controls for sterility (RPMI 1640 medium without inoculum), growth (inoculum suspended in RPMI 1640 without antifungal agents) and activity of other drugs used in the study (concurrent testing of a pan-susceptible yeast) were included in experiments. The minimal inhibitory or minimal effective concentration values for RP557 were assessed visually, with inhibition endpoints determined in comparison to the growth control.

 Minimum fungicidal concentrations (MFCs) were determined in instances where MICs were deemed low and killing seemed possible. MFCs were determined by plating 25% of the culture volume, from the lowest drug concentration without visible growth, onto agar to ascertain the lowest concentration capable of killing 99% of the inoculum.

### Antimicrobial peptide RP557 in interaction with antifungal drugs

To evaluate the combination activity of RP557 with antifungal drugs, a checkerboard assay was performed, determining the fractional inhibitory concentration index (FICi) [20]. The selection of the commercial drugs for these studies was based on the known clinical utility of those drugs as single agents against the individual pathogens. A total of 20 isolates were selected, including 11 *Sporothrix* spp. (*S. brasiliensis*, *n*=5; *S. schenckii*, *n*=5; and *S. globosa*, *n*=1) for the combination of RP557 with ITZ; 6 *Candida auris* isolates tested in combination with AMP RP557 plus MYC, CAS, AmB or FCZ; and 3 *A*. section *Fumigati* isolates tested in combination with RP557 plus MYC, VCZ or AmB. The endpoint was 100% inhibition for AmB or ITZ and 50% for other combinations. Interaction effects were categorized as strong synergism (FICi <0.5), weak synergism (0.5<FICi<1), additive (1<FICi<2), indifference (FICi=2) or antagonism (FICi >2) [20].

## Results

### Pathogenic yeasts

RP557 inhibited all *Cryptococcus neoformans* isolates (*n*=5) with MICs narrowly ranging from 4 to 8 µg ml^−1^. MICs for *Candida auris* isolates (*n*=6), by contrast, ranged from 4 to >64 µg ml^−1^ (Table 1).

**Table 1.** *In vitro* inhibitory and fungicide activity of antimicrobial peptide RP557 against fungal pathogens

Fungus	Isolate ID	RP557 MIC*	RP557 MFC†
*Candida auris*			
	6119	≥64	--
	6125	≥64	--
	6126	≥64	--
	20-188	16	--
	20-191	16	--
	20-253	4	--
*Cryptococcus neoformans*			
	421	4	--
	1225	8	--
	1346	4	--
	1871	4	--
	2215	4	--
*Aspergillus* section *Fumigati*			
	1270	>64	--
	1666	>64	--
	3093	>64	--
	13-21	>64	--
	14-17	>64	--
	15-31	>64	--
	15-55	>64	--
	15-77	>64	--
	15-80	>64	--
	16-120	>64	--
	17-67	>64	--
	18-03	>64	--
	18-25	>64	--
*Aspergillus* section *Nigri*			
	5008	32	--
	5020	32	--
*Aspergillus* section *Flavi*			
	23-24	64	--
	23-25	64	--
*Fusarium* sp.			
	07-136	>64	--
	07-144	16	--
	12-22	8	--
	18-73	16	--
	23-28	2	--
	96-1	8	--
*Trichophyton mentagrophytes*			
	6895	8	>32
	6959	8	16
	8113	8	32
	8910	2	8
*Trichophyton rubrum*			
	9540	4	32
*Mucorales*			
	13-91	32	
	16-88	16	
	16-121	>64	
	16-129	32	
	17-102	16	
	23-26	64	
	23-27	2	
*Phialophora verrucosa*		1	2
*Cladophialophora carrionii*		1	1
*Fonsecaea pedrosoi*		1	4
*Madurella mycetomatis*		0.5	1
*Sporothrix brasiliensis*			
	716	16	
	1078	8	
	1878	32	
	2691	8	
	3357	>64	
	3952	>64	
	4559	16	
	4720	32	
	4748	>64	
	5150	8	
	9011	>64	
	9012	>64	
	9013	16	
	9014	16	
	9015	8	
	9016	16	
	9017	>64	
	9018	2	
	9019	8	
	9020	4	
*Sporothrix schenckii*			
	1917	4	
	2657	4	
	5196	2	
	7453	8	
	11516	16	
*Sporothrix globosa*			
	5823	8	

*Minimal inhibitory concentration (MIC) values for RP557 with 50% inhibition endpoint determined in comparison to the growth control.

†MFC: minimum fungicidal concentration (MFC) of RP557 defined as the lowest concentration capable of killing 99% of the inoculum. Isolate ID: Isolate identification. Values expressed as µg/mL.

Synergy with echinocandins was shown against 100% of the *Candida auris* isolates (*n*=6) and with AmB against 83% (5/6). An indifferent effect was observed combining RP557 with FCZ against *Candida auris* isolates tested (*n*=3, and thus not pursued further). No antagonistic effect occurred (Table 2) with any of these major antifungals.

**Table 2.** *In vitro* results of antimicrobial peptide RP557 interaction with antifungal drugs against fungal pathogens

Fungus	Isolate ID	RP557 MIC* alone	Second drug MIC alone		FICi†	Effect
*Candida auris*						
	6119	≥64	MYC	0.25	≤0.5	WS
			AmB	2	2	IND
	6125		≥64	MYC	0.12	≤0.75	WS
			AmB	2	≤0.52	WS
	6126	≥64	MYC	0.12	≤0.52	WS
			AmB	1	≤0.52	WS
	20-188	16	CAS	0.09	≤0.13	SS
			AmB	1	≤0.38	SS
			FCZ	>64	2	IND
	20-191	16	CAS	0.05	≤0.28	SS
			AmB	1	≤0.28	SS
			FCZ	>64	2	IND
	20-253		4	CAS	>6.25	≤0.13	SS
			AmB	0.5	≤0.38	SS
			FCZ	>64	2	IND
*Aspergillus* section *Fumigati*						
	1270	>64	MYC	≤0.03	2	IND
			VCZ	0.5	2	IND
			AmB	1	≤0.508	WS
	1666	>64	MYC	≤0.03	2	IND
			VCZ	0.5	2	IND
			AmB	1	2	IND
	3093	>64	MYC	≤0.03	2	IND
			VCZ	0.5	2	IND
			AmB	1	≤0.508	WS
*Sporothrix brasiliensis*						
	1078	8	ITZ	0.25	2	IND
	4559	16		>8	≤0.28	SS
	5150	8		0.5	1	AD
	9014	16		4	≤0.38	SS
	9016	16		0.125	≤0.5	WS
*Sporothrix schenckii*						
	1917	4	ITZ	8	≤0.19	SS
	2657	4		>8	≤0.63	WS
	5196	2		8	≤0.56	WS
	7453	8		8	≤0.5	WS
	11516	16		0.5	≤0.63	WS
*Sporothrix globosa*						
	5823	8	ITZ	>8	≤0.28	SS

*Minimal inhibitory concentration (MIC) values for RP557, caspofungin (CAS), micafungin (MYC), voriconazole (VCZ), and fluconazole (FCZ) with 50% inhibition endpoint determined in comparison to the growth control, and MIC values to amphotericin B (AmB) and itraconazole (ITZ) with 100% inhibition endpoint. Values expressed as µg/mL.

†Fractional inhibitory concentration index (FICi) determined to evaluated the effects of drug combinations, categorizing as strong synergism (SS) (FICi < 0.5), weak synergism (WS) (0.5 < FICi < 1), additive (AD) (1 < FICi < 2), indifference (IND) (FICi = 2), or antagonism (ANT) (FICi > 2) (Poester et al., 2021). Isolate ID: Isolate identification.

### Pathogenic mycelial fungi

Inhibitory activity of RP557 against *A*. section *Fumigati* (*n*=13) was not detected (MIC >64 µg ml^−1^) (Table 1). However, *A*. section *Nigri* (*n*=2) and *A*. section *Flavi* (*n*=2) were inhibited at concentrations ranging from 32 to 64 µg ml^−1^. In the interaction analysis, two of three isolates showed a weak synergistic effect in combination with AmB, while in all others, RP557 efficacy was indifferent to AmB, MYC or VCZ (Table 2).

Eighty-three percent (five of six) of *Fusarium* spp. isolates were inhibited by RP557, with MICs in those ranging from 2 to 16 µg ml^−1^, and MIC values for *Mucorales* ranged from 2 to 64 µg ml^−1^ (*n*=6), with only one isolate not inhibited by RP557.

### Fungi causing skin and soft tissue infection

These pathogens were also inhibited *in vitro* by RP557. Dermatophyte MICs (*n*=5) were between 2 and 8 µg ml^−1^, and 80% of the isolates were also killed by the peptide, with MFC values ranging from 8 to 32 µg ml^−1^.

Chromomycosis and mycetoma pathogens (*n*=4) showed the lowest MIC values for RP557 susceptibility, with MICs ranging from 0.5 to 1 µg ml^−1^ and MFC values from 1 to 4 µg ml^−1^ (Table 1).

### *Sporothrix* spp.

Seventy-seven percent (*n*=26) of *Sporothrix* spp. isolates had MIC values ranging from 2 to 32 µg ml^−1^, whereas six of these isolates (23%) (all *S. brasiliensis*) were not inhibited by the peptide (Table 1). Regarding the interaction with ITZ, 91% of 11 isolates showed a synergistic (strong, *n*=4; weak, *n*=5) or additive (*n*=1) effect when combined with the peptide (Table 2). No antagonism was seen.

## Discussion

Here, we conducted an *in vitro* study to evaluate the inhibitory and/or fungicidal activity of the synthetic peptide RP557 against fungal pathogens. In agreement with data from a previous study of this compound against *Candida* species [16], we showed that the RP557 peptide has the potential to be used in the development of a new antifungal and/or a new co-adjuvant therapy for fungal infections, showing a broad-spectrum activity, effective against yeasts, moulds and even dimorphic fungi. Out of a total of 76 isolates tested, the peptide demonstrated some inhibitory activity against two-thirds of fungal pathogens. The overall impression of RP557 tested alone is a robust activity against *Cryptococcus neoformans*, agents of chromomycosis and mycetomas, dermatophytes, *Fusarium* spp. and *Sporothrix* spp. Lesser effectiveness was observed against *Mucorales*, *Candida auris* and *Aspergillus* spp. However, some promising drug interactions in efficacy against *Sporothrix* spp. and *Candida auris* were noted.

 RP557 was able to inhibit and kill agents of chromomycosis and mycetomas, in low concentrations (1 and 4 µg ml^−1^, respectively). Chromoblastomycosis and eumycetoma are disfiguring subcutaneous implantation mycoses primarily found in tropical and subtropical regions and included in the roadmap for neglected tropical diseases by the WHO [7]. They represent a global health concern, mainly associated with poverty, and lead to appreciable morbidity in affected patients [21,22]. One of the main challenges in treating these diseases is the prolonged oral antifungal therapy required, typically with itraconazole or terbinafine, which often leads to adverse effects [23,25]. Thus, RP557 could be a promising alternative for both mycoses. In this regard, further studies should explore topical therapies for subcutaneous mycoses and dermatophytes – the latter a group also inhibited in our study by the peptide – that cause skin mycoses with a worldwide impact on the quality of life of millions of patients [5]. Topical therapy in other contexts appears promising [26,29]. This approach would provide local concentrations exceeding even our high MICs, potentially offering more effective treatment options. Oral administration should be explored too, but may be defeated by the susceptibility of peptide degradation in the gastrointestinal environment. Recent studies have suggested methods to improve RP557 delivery by several routes, reduce enzymolysis and reduce adverse effects [30]. Efficacy of RP557 against other pathogens [26,28, 29, 31] is consistent with a mechanism involving rapid action to target cell walls (possibly electrostatically) to attack cell membranes, producing lysis and concomitantly reducing inflammation. This multi-microbial activity suggests the need for further RP557 clinical studies and clinical development.

 Similarly, RP557 is a potential treatment for sporotrichosis, a global subcutaneous mycosis and an emerging zoonotic disease associated with the transmission of *S. brasiliensis*, especially in Brazil – the epicentre of this zoonotic disease – and in South America [9,32]. One of the main steps to controlling the dissemination of sporotrichosis is ensuring effective treatment for hosts, primarily cats but also humans [9,33]. Our study found the useful activity of RP557, most notably in combination with itraconazole (ITZ) (91% beneficial effect), which is the first-line drug for treating sporotrichosis [24]. Interestingly, this association could reclassify some *Sporothrix* isolates that were non-wild-type for ITZ (high MIC) as being wild-type (MIC <4 mcg ml^−1^). Therefore, we highlight the potential of this peptide for use in combination therapy with ITZ, emphasizing the need for further studies on this interaction in animal models using an oral and topical approach.

Topical therapy for cutaneous and subcutaneous mycoses would have potential advantages in some humans, who may be reluctant to take pills, and especially for animals (e.g. feline victims of sporotrichosis), where oral administration of drugs may be more problematic. Antifungal efficacy in topical therapy with AMPs has already been demonstrated [34]. The broad antimicrobial activity of these AMPs, in addition to their apparent immunomodulatory properties, might particularly lend itself to topical therapy, or prophylaxis, for burns or trauma wounds; polymicrobial invasion is always a threat in these situations. Present RP557 research activities include a focus on demonstrating efficacy in the empiric treatment of burn infections.

 Another fungal pathogen for which RP557 enhanced the antifungal activity of current drugs was *Candida auris*. The peptide appreciably improved the activity of echinocandins and AmB in the majority of isolates. *Candida auris* is a global concern due to the severity of clinical manifestations, its resistance to available drugs and its high potential to cause outbreaks [35]. Our results support further investigation of RP557 as a potential candidate for the future treatment of candidiasis, including cases caused by multidrug-resistant *Candida auris*. These findings align with the previous results of Woodburn *et al.* [16], who demonstrated that RP557 does not induce resistance upon repeated exposure in *Candida tropicalis*. This suggests a lack of resistance development to the peptide; however, further *in vitro* and *in vivo* studies with *Candida* species are necessary to confirm this hypothesis.

 Regarding the antifungal activity of antimicrobial peptides, studies indicate that these peptides can disrupt the plasma membrane by binding to sterols, or interfere with ribosomal subunits, thereby affecting DNA, RNA, protein and other macromolecule synthesis, as well as intracellular respiration [36,37]. AMPs have been demonstrated to have the following effects on microbes and in infected animals: on reactive oxygen species, nucleic acids, iron and calcium metabolism, cell signalling, cell death pathways and the proteome, including cellular enzymes and protein folding. Such effects could contribute to antifungal activity *in vitro* and *in vivo* [11,12, 34, 38,49]. A particularly interesting finding regarding AMPs as antifungals is evidence of their binding to cell wall glucan, suggesting a possible role in inhibiting cell wall synthesis and/or degradation – similar to the mechanism of echinocandins, which are semi-synthetic derivatives of natural peptides [50]. Echinocandins inhibit *β*(1,3)-d-glucan synthase, leading to the disruption of fungal cell wall synthesis [46,51]. The best direct evidence we have for RP557 antifungal mechanism is that RP557 appears to disrupt fungal membranes [16]. The proposed mechanisms of AMPs may complement each other, working synergistically either as a stand-alone treatment or in combination with other antifungal drugs, ultimately inhibiting or killing fungal cells. This supports further investigation into RP557 as a potential antifungal candidate, especially considering the typically low toxicity of drugs targeting the fungal cell wall [52]. In this context, a previously mentioned study suggests that RP557 exhibits limited toxicity to mammalian cells, as assessed through bioluminescence and lactate dehydrogenase membrane disruption assays [16]. The 10% lethal dose (LD10) for human keratinocytes was estimated at 287 µg ml^−1^ – considerably higher than the *in vitro* tested range here (0.5 to 64 µg ml^−1^) [16]. These findings support the peptide’s potential safety for antifungal use and highlight the need for further studies to evaluate high-dose RP557 in animal models to determine its toxic dose and overall safety profile.

 Some limitations of this study include the absence of *in vivo* efficacy data, particularly correlations between the MICs demonstrated and *in vivo* effective doses. The number of interaction tests needs expansion for several of the species tested here. More MFC data for some pathogens, and more drug–drug interaction in inhibition and killing studies would be desirable. Speciation of the *Mucorales*, to look for species-specific differences, would be of interest. Also of interest would be an enlarged study of *Candida auris*, including representatives of all clades, beyond the isolates already included [17]. The reason for the variation in susceptibility between the various fungal species in our study is unknown. The mechanisms of action could be different in different species, owing to the diversity of the targets among fungal species, or differing abilities of RP557 to reach its membrane target, owing to different compositions of cell walls in different species. However, our study represents a crucial first step in demonstrating the broad antifungal spectrum of RP557, beyond the previously published studies with *Candida* species [16]. It paves the way for future research to explore each pathogen and mycosis in greater depth, assess their *in vivo* susceptibility to RP557 and investigate the peptide’s precise mechanism of action in different fungal species. Other AMP molecular manipulations may be of interest in future studies.

 In conclusion, we present an extensive survey of a synthetic derivative of natural peptide molecules, demonstrating a broad spectrum of antifungal activity against WHO priority pathogens and some neglected ones. Further studies should continue with *in vitro* tests, study tissue penetration and toxicology and assess the efficacy of RP557 *in vivo* for all the fungi included in our study, even those with lesser *in vitro* activity (such as *Aspergillus* spp. and *Mucorales*). It is important to consider that the peptide may contribute to innate and natural immunological modulation, which could, in turn, support the resolution of fungal infections, and this avenue of action might make the absolute MICs discovered here less critical.

## Abbreviations

AmB: amphotericin B
AMP: antimicrobial peptide
CAS: caspofungin
CLSI: Clinical and Laboratory Standards Institute
FCZ: fluconazole
FICi: fractional inhibitory concentration index
ITZ: itraconazole
MFCs: minimum fungicidal concentrations
MYC: micafungin
VCZ: voriconazole
WHO: World Health Organization
